# The Impact of Near Vision Impairment on Activities of Daily Living Across the Life Course

**DOI:** 10.1097/APO.0000000000000472

**Published:** 2022-01-18

**Authors:** Julie-Anne Little, Bruce Moore, Nathan Congdon

**Affiliations:** Centre for Optomtry and Vision Science, Biomedical Sciences Research Institute, Ulster University, Coleraine, UK; New England College of Optometry Boston, MA, US; School of Medicine Dentistry and Biomedical Sciences, Queen’s University Belfast, Belfast, UK Orbis International, New York, US Zhongshan Ophthalmic Center; and Sun Yat-sen University, Guangzhou, China

## Editorial - Blind

He et al’s report^1^ from a multinational population-based cohort underscores the fact that near vision impairment from presbyopia has its onset and peak during the heart of the working years. The importance of this for poverty-alleviation strategies has been highlighted by the PROSPER trial,^2^ in which provision of near vision correction to Indian tea-pickers was shown to have a larger effect on work productivity than previously reported for any other health intervention in a lower-middle income country (LMIC) setting. The on-going PROSPER II and III trials (NCT04629820 and NCT04654013) are now designed to extend the evidence base from agriculture into other work settings such as garment manufacture, and to asses the impact of presbyopia correction on workplace retention in the elderly. This latter is of particular importance due to declining workforce participation seen in the fifth decade of life and beyond in many LMICs, as shown in Key Indicators of the Labour Market data from the Internaional Labour Organization.

The manuscript by Cherwek et al^3^ in this issue shows that near vision impairment (NVI) is already apparent in 20% to 25% of female garment workers in Bangladesh aged 30 to 35 years, and these persons with NVI were earning US $10 less per month, a finding that remained significant when adjusting for other key predictors of income. The early onset of NVI in these workers, and the higher prevalence among rural as compared to urban workers in this setting, appears to be due to hyperopia, and is consistent with the finding of higher myopia prevalence in more educated, urban cohorts.^4^ This highlights the important possibility that hyperopia and associated earlier onset of presbyopia, with consequent lower earnings, may be another unfair economic burden borne by rural dwellers.

Hyperopia is the refractive error which potentially has its greatest adverse effect on near viewing and is most critical to learning by reading and to near work.^5,6^ Despite the traditional view that children have large accommodative reserves and that uncorrected or undercorrected hyperopia is relatively benign in the absence of strabismus, evidence exists that children who have uncorrected or undercorrected hyperopia have lower educational scores.^7^ Due to the extra accommodative effort required for near work, uncorrected hyperopia places a particular burden on clarity and sustainability of near vision.^6^ This additional effort required by children with hyperopia may lead to the experience of fatigue when carrying out close work, which may lead in turn to increased inattention and poor classroom behaviour. Recently, the work of the VIP-HIP group^5^ in the United States has demonstrated that hyperopia is associated with lower reading ability and educational achievement; investigators found that many children with hyperopia at a level that could benefit from spectacle correction did not have glasses. However, to date, the causal structure underpinning the relationship between hyperopia and educational achievement remains incompletely understood. The metaanalysis conducted in the systematic review^8^ in this issue reveals that uncorrected hyperopia is associated with poorer academic performance. So, while we are addressing the knowledge gap regarding the impact of uncorrected hyperopia on academic achievement, their remains much work to be done in the form of high-quality clinical trials to see whether correction of hyperopia improves academic outcomes.

Taken together, the above evidence suggests a continuum of NVI and associated loss of educational and economic opportunities across the life-course. Whether it is a hyperopic child experiencing reading and learning difficulties, an older presbyopic adult no longer able to work, read, recognise money,^9^ or use a smartphone,^10^ or a worker in her early 30s suffering from reduced income and productivity from a combination of hyperopia and early-onset presbyopia, the situation is the same: the inability to carry out important life tasks due to an accommodative reserve that is not adequate for the job at hand without correction.

More data are needed to assess the benefits of correcting NVI in both children and adults. But it would appear that school vision screening models have neglected for too long assessment of near vision, and that more must be learned about the true prevalence of hyperopia in children and hyperopia-induced early-onset presbyopia in young adults, particularly in LMICs. There is a need to validate simpler screening protocols for childhood hyperopia appropriate for low-resource settings, and to further explore, initially through cross-sectional studies, the association between hyperopia and markers of poor reading and learning.

If such studies suggest that hyperopia is prevalent in LMIC settings such as Africa with low rates of myopia, and if appropriate detection methods are found, together with evidence of a cross-sectional association between hyperopia and reduced reading levels, trials are warranted to determine if hyperopic correction can improve learning outcomes. We are increasingly confident that the answer is “yes” regarding work productivity and correction of NVI among adults. The question of hyperopia’s impact on children’s learning is equally deserving of our attention.

The Human Development Index (HDI) championed by the United Nations Development Programme highlights three basic measures of wellbeing: life expectancy, literacy, and per capita GDP. It increasingly appers that vision is a necessary ingredient in each of these, to give all persons equal opportunities “to thrive, flourish and achieve their full potential at all stages across life course”.
